# Feasibility of the Energy Expenditure Prediction for Athletes and Non-Athletes from Ankle-Mounted Accelerometer and Heart Rate Monitor

**DOI:** 10.1038/s41598-020-65713-7

**Published:** 2020-06-01

**Authors:** Chin-Shan Ho, Chun-Hao Chang, Yi-Ju Hsu, Yu-Tsai Tu, Fang Li, Wei-Lun Jhang, Chih-Wen Hsu, Chi-Chang Huang

**Affiliations:** 1 Graduate Institute of Sports Science, National Taiwan Sport University, Taoyuan, Taiwan; 2 Department of Physical Medicine and Rehabilitation, Taipei City Hospital, Zhongxiao Branch, Taipei City, Taiwan

**Keywords:** Weight management, Biomedical engineering

## Abstract

Due to the nature of micro-electromechanical systems, the vector magnitude (VM) activity of accelerometers varies depending on the wearing position and does not identify different levels of physical fitness. Without an appropriate energy expenditure (EE) estimation equation, bias can occur in the estimated values. We aimed to amend the EE estimation equation using heart rate reserve (HRR) parameters as the correction factor, which could be applied to athletes and non-athletes who primarily use ankle-mounted devices. Indirect calorimetry was used as the criterion measure with an accelerometer (ankle-mounted) equipped with a heart rate monitor to synchronously measure the EE of 120 healthy adults on a treadmill in four groups. Compared with ankle-mounted accelerometer outputs, when the traditional equation was modified using linear regression by combining VM with body weight and/or HRR parameters (modified models: Model A, without HRR; Model B, with HRR), both Model A (*r*: 0.931 to 0.972; ICC: 0.913 to 0.954) and Model B (*r*: 0.933 to 0.975; ICC: 0.930 to 0.959) showed the valid and reliable predictive ability for the four groups. With respect to the simplest and most reasonable mode, Model A seems to be a good choice for predicting EE when using an ankle-mounted device.

## Introduction

Lightweight and easy-to-wear designs have been widely adopted to increase the efficiency of wearable inertial sensor technology and to optimize their economic benefits, resulting in the popularization of applications in sports science, health care, and other related fields^1,2^. The use of wearable devices requires accurate signal collection and analysis as the accuracy of exercise intensity or volume analysis can affect a coach’s or user’s judgments of training effects and the range of adjustments to the training plan, particularly the monitoring of energy expenditure (EE) during training exercises. To control the training volume and maintain the energy balance, whether EE is consistent with energy intake is the key to enhancing sports performance^3,4^.

The difference between athletes’ and non-athletes’ resting metabolic rates (RMRs) and total energy expenditure (TEE) has been proven by researchers through measurements of golden standards (indirect calorimetry or doubly labelled water)^3,5,6^. Studies have also shown that total muscle mass is proportional to oxygen uptake^7,8^. Statistically, the athlete’s muscular system is well developed, and even the vital organs (such as the liver, heart, and kidneys) are larger than average. Because of this, athletes have a higher EE level^9^. From the perspective of body weight management, inaccurate EE measurements can cause undernutrition or overnutrition, increase the risk of changing the muscle-to-fat ratio, affect sports performance, and even produce adverse health effects^10,11^. Therefore, a more accurate measurement technology is needed to monitor the EE of groups with different physical fitness levels. The accelerometer, known primarily as a physical activity monitoring device, has attracted attention with respect to its accuracy and scope of applications.

The three common wearing positions of physical activity monitoring devices are the wrist, hip, and ankle^12–14^. Many studies have reported the reliability and validity of EE measurements of devices worn in different locations^15–18^. The EE estimation equation is based on the experimental parameters of the treadmill and daily activity tests in a laboratory or free-living environment, and the subsequent calculation is based on the tri-axial (three-dimensional) vector magnitude (VM) activity counts produced during exercise^12,19,20^. Apart from the EE estimation equation for the general population, EE estimation equations for specific groups, such as children or adolescents^21–23^, elderly people^24^, and users in wheelchairs^25,26^, have been developed. According to the relevant references for monitoring physical activity, few EE estimation equations are ideal for athlete groups. Therefore, the uses of the equations are restricted from some perspectives.

Due to the nature of micro-electromechanical systems (MEMS), the VM activity counts of an accelerometer can, under the same exercise intensity, vary depending on the wearing position, and without an appropriate EE estimation equation, bias can occur in the measured values. Many accelerometer-type wearable inertial sensors on the market are wrist- or hip-mounted, but fewer are placed on the ankle or foot. The manual of the research-level ActiGraph accelerometer (Actigraph Corporation, Pensacola, FL, USA) suggests that users wear the device on the wrist or waist. Some products, such as the Nike+ sensor (Nike Inc, OR, USA) and the Adidas miCoach Footpod sensor (Adidas AG, Germany), are foot-mounted, and their EE values are measured mainly based on accelerometer signals and the distance recorded by a global positioning system (GPS). The foot-mounted device still has commercial market demand. According to the research, foot and ankle devices can overestimate EE, resulting in inaccurate results^18,27^.

In short, to overcome the insufficient accuracy of accelerometer-based wearable devices, some researchers proposed individual and wearing position calibrations using physiological parameters (such as heart rate, HR) to enhance the reliability and validity of EE estimates^28,29^. Our previous studies showed that including heart rate reserve (HRR) parameters in the prediction equation can further improve the reliability and validity of EE estimates, especially for individual physical fitness and in various wearing positions^30,31^. To meet the needs of various wearing positions, we modified the traditional EE prediction equation (Freedson VM3 combination equation, 2011)^32^ for wearing devices on wrist and hip, making it suitable for ankle-mounted accelerometer devices. To increase utility, we collected users of four different physical groups. Therefore, we presumed that the HRR parameter is an important indicator for calibrating the physical fitness of different groups, can be used to calibrate the bias of EE estimated by ankle-mounted devices, and can further enhance the reliability and validity of EE. In this study, we aimed to include HRR parameters in the EE prediction equation as a calibration indicator to adjust the EE prediction equation primarily for ankle-mounted devices among four groups, which included non-endurance athlete, endurance athlete, sedentary, and exercise-habit groups.

## Methods

### Study design

We employed indirect calorimetry as a criterion measure (CM) and an ankle-mounted accelerometer monitoring device to measure the EE of four representative groups: athletes (non-endurance group and endurance group) and non-athletes (sedentary group and exercise-habit group). An accelerometer-based HR monitoring device was simultaneously used to measure their HR during the exercise tests (treadmill speed: 4.8, 6.4, 8.0, 9.7, and 11.3 km/h). Because HRR can directly reflect the physical fitness level of the participant, we adopted the HRR parameter as a correction factor for the prediction equation to increase the accuracy of the EE predicted. From these measurement periods, linear regression equations were determined for each physical fitness level based on average accelerometer VM, body weight (BW), and without/with HRR parameters (i.e., Model A and Model B, respectively), and the corresponding EE measured by indirect calorimetry. We explored whether modifying the traditional EE prediction equation (Freedson VM3 combination equation, 2011)^32^ would be ideal for ankle-mounted devices with users of different fitness levels. The study procedures were approved by the Institutional Review Board of Fu Jen Catholic University (New Taipei City, Taiwan) and informed consent was provided by all participants before the experiment. This study was conducted in full conformance with the relevant guidelines and regulations, i.e., the principles of *the Declaration of Helsinki* guidelines. The study was registered at http://irb.rdo.fju.edu.tw/ (reference number: C106056). We recruited volunteers with four kinds of physical fitness characteristics using an open, independent, and random method.

### Participants

A total of 120 adults, non-athletes and athletes, voluntarily participated in this study. The participants were divided into four groups of 30 people each according to their physical fitness. Non-athletes were divided into a sedentary group (SG) (male: 40.0%, female: 60.0%) and an exercise-habit group (EHG) (male: 46.7%, female: 53.3%) according to their daily living and exercise habits. Athletes were divided into a non-endurance group (NEG) (male: 56.7%, female: 43.3%) and an endurance group (EG) (male: 63.3%, female: 36.7%) according to their specialties. The SG was defined as participants without any regular exercise habits, or time spent is sitting or lying down. The EHG was defined as participants who regularly exercised at least three days per week with moderate-intensity physical activity each time. The NEG comprised non-endurance athletes who were competitors in intercollegiate games and engaged in routine training at least 5 times per week in a specific program, with a focus on sprint races and throwing sports. The EG was composed of endurance athletes who were competitors in intercollegiate games and participated over 5 times each week in routine training in a specific program, with a focus on middle- or long-distance races. Only the volunteers with a good health condition were recruited for this study. Due to some conditions could prevent volunteers from completing the test safely, such as exercise contraindications, taking drugs that could affect the metabolic rate or cardiovascular disease. During the treadmill test, participants were equipped with experimental devices, including mask, accelerometer, and heart rate monitor in a laboratory with an average room temperature of 23 °C. The participants’ anthropometric measurements and body composition features are listed in Table 1.

**Table 1:** Anthropometry and body composition characteristics of participants.

	SG	EHG	NEG	EG	p*
Age (years)	21.9 ± 1.9	21.7 ± 1.6	21.1 ± 1.7	20.9 ± 1.7	0.118
Height (cm)	166.9 ± 8.1	167.3 ± 8.5	171.2 ± 7.7	170.0 ± 5.8	0.078
Sex	12 males, 18 females	14 males, 16 females	17 males, 13 females	19 males, 11 females	
Training time (h/week)	~ 0	1.5 ~ 5	27.2 ± 2.2	27.8 ± 1.5	
Body weight (kg)	67.2 ± 13.9^ b^	64.6 ± 12.6	68.7 ± 16.9 ^b^	59.8 ± 8.0	0.057
BMI (kg/m^2^)	23.7 ± 3.5 ^b^	23.1 ± 3.3 ^b^	23.3 ± 4.7 ^b^	20.8 ± 2.1	0.010
Skeletal muscle mass (kg)	26.6 ± 5.7 ^a^	28.4 ± 6.0 ^a^	32.1 ± 7.1	29.1 ± 4.5	0.005
Percentage of body fat (%)	19.1 ± 8.5 ^b^	20.2 ± 6.6 ^a,b^	16.3 ± 5.4	8.4 ± 3.8 ^a^	<0.001
RMR (kcal/day)	1414.1 ± 200.7 ^a^	1464.2 ± 206.9 ^a^	1591.1 ± 250.6	1490.0 ± 160.0	0.011

Values are reported as mean ± SD. SG, sedentary group. EHG, exercise-habit group. NEG, non-endurance group. EG, endurance group. * *p*-value was calculated from one-way ANOVA test among four groups. BMI, body mass index. RMR, resting metabolic rate. ^a^ Significantly different from NEG, *p* < 0.05. ^b^ Significantly different from EG, *p* < 0.05.

### Anthropometric Measurements and Body Composition

Anthropometric measurements and body composition features were assessed through standard procedures. Each participant’s height, body weight, and body composition were measured with the InBody 570 Body Composition Analyzer (Biospace, Inc. Seoul, Korea). The InBody 570 is a multi-frequency bioelectrical impedance analyser (MFBIA). This is a well-established method to determine body composition, such as body weight, body mass index (BMI), skeletal muscle mass, percentage of body fat, and resting metabolic rate (RMR). All participants wore light sportswear, removed metal jewelry, shoes and socks, and stood on the machine barefoot to minimize possible errors.

### Measurement of EE by Indirect Calorimeter and Accelerometer Method

For the indirect calorimeter method, the Cardiopulmonary Exercise Testing System (Vmax Encore 29 System, VIASYS Healthcare Inc., Yorba Linda, CA, USA) was employed to conduct the metabolic CM. Warming up of the Vmax system for at least 15 minutes and the calibration should be done before each test. Participants were equipped with a well-fitted, noses and mouths covering mask (Hans Rudolph Inc., Kansas City, MO, USA) which connected to flow sensor and sampling line was to collect the amount of oxygen consumption (VO_2_) and carbon dioxide output (VCO_2_) they breathed (breath by breath).

For the accelerometer method, all the test data was collected by an ActiGraph GT9X-Link (Actigraph Corporation, Pensacola, FL, USA), a tri-axial accelerometer (size: 3.5 × 3.5 × 1 cm, weight: ~14 g). The initialization of the ActiGraph accelerometer and chest mount Polar H10 HR monitor (Polar Electro Oy, Kempele, Finland) was done by ActiLife6 software (version 6.12.1, ActiGraph, Cary, NC, USA). In this study, the sampling frequency of the accelerometer and heart rate monitor was set to 30 Hz, and the physical activity parameters (i.e., calories, and VM), and HR data were collected in 10-second intervals set manually via ActiLife6 software. The ActiGraph accelerometer was attached to their right ankle, and the participants took one test at a time.

### Treadmill Test

The setting in the laboratory, the participants were requested to complete treadmill (h/p cosmos mercury 4.0, Nussdorf-Traunstein, Germany) exercise tests (from walking to running) at speeds of 4.8, 6.4, 8.0, 9.7, and 11.3 km/h. Before the experiment started, the resting heart rate (*HR*_*rest*_) in the sitting position was first measured. The participant sat in a resting position for 20 min, and the lowest HR recorded during the last 5 min was set as the resting value^30^. Each test took at least 3 minutes, and tests at different speeds were divided by two-minute breaks in an approach modified from Tudor-Locke *et al*.^33^. The indirect calorimetry, HR monitor, and accelerometer outputs for all tests were synchronously and continuously recorded during the process. If a participant failed to keep pace with the treadmill speed, heart rate exceeded the safety range (220 bpm – age) or gave up midway, the experiment data of this test and any personal information would be erased.

### Data Analysis

**Figure 1:**
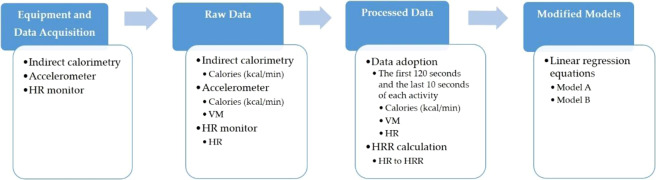
Data process diagram. VM, vector magnitude. HR, heart rate. HRR, heart rate reserve. BW, body weight.

All 120 participants completed the exercise test, and all indirect calorimetry (Vmax system), HR monitor, and accelerometer (ActiGraph GT9X-Link) data were exported into Microsoft Excel (Excel version in Microsoft Office 2013 for Windows). The Vmax system, HR monitor data, and the ActiGraph GT9X-Link accelerometer data were used to calculate the time-series parameters of the 10 × 10 s block. According to Lyden *et al*.^20^, in the treadmill exercise test, the participant performs in unsteady and steady states during each specific speed interval, which means that the acquired raw data consist of unsteady and steady data, of which, steady data is considered valid. Therefore, the first 120 seconds of each activity were deleted to ensure that the participant’s exercise intensity level had reached the steady state and the last 10 seconds were eliminated to minimize any researcher error in timing synchronization between the accelerometer and the metabolic measurements. After the elimination of the first 120 seconds and the last 10 seconds, we required at least 30 s of the remaining data for inclusion in the analyses. The data process is shown in Fig. 1.

### Indirect Calorimetry

The VO_2_ and VCO_2_ values for each walking/running trial were used to calculate EE as a continuous summed value in calories (Kcal/min) to determine criterion measure EE (CM-EE) using Weir’s equation^34^, which considers the energy derived from different fuel sources. The Eq. (1) is as follows:

1 $${\rm{CMEE}}({\rm{Kcal}}/\,{\rm{\min }})=3.491\times V{O}_{2}+1.106\times VC{O}_{2}$$

where VO_2_ is oxygen consumption in liters per minute, and VCO_2_ is the rate of carbon dioxide production in liters per minute.

Body weight, height, and age were used to calculate resting metabolic rate (RMR) for each participant^35^. This RMR value was then subtracted from their total EE value for each walking/running trial, providing a final value for physical activity-related EE^36^.

### Accelerometer

The ActiGraph GT9X-Link data (GT9X-EE) were analyzed in ActiLife6 software, and the GT9X-EE was calculated for each walking/running trial using the prediction equation which developed by Sasaki *et al*.^37^, labeled “Freedson VM3 Combination equation (2011)”^32^ in the Actilife software. This equation uses the three-dimensional (3D) vector magnitude (VM) (i.e., a combination of the tri-axial accelerometer data) to predict calories (kcal/min). The Eq. (2) is as follows:

2 $${\rm{GT}}9{\rm{X}}-{\rm{EE}}({\rm{kcal}}/\,{\rm{\min }})=0.001064\times VM+0.087512\times BW-5.500229$$

where the 3D ${\rm{VM}}=\sqrt{{({\rm{axis}}1)}^{2}+{({\rm{axis}}2)}^{2}+{({\rm{axis}}3)}^{2}}$ according to 10 s epoch lengths analyzed in ActiLife6 software, and BW is body weight (kg).

All the EE values were divided by the body weight for standardization and are presented in kcal/kg/min. HRR = *HR*_*max*_ − *HR*_*rest*_ refers to the difference in *HR*_*max*_ (each test stage) and *HR*_*rest*_, where *HR*_*max*_ is the maximum heart rate at each test stage, and *HR*_*rest*_ is the pre-test measure of resting HR.

### Statistical Analysis

All statistical data of this study that be adopted was analyzed via SPSS Statistics version 20 statistical software (IBM Corp., New York, NY, USA). The significance level was set to *α* = 0.05. All data were represented in the form of mean ± SD. The comparisons of the fundamental data and body compositions of the four groups of participants were conducted via One-way analyses of variance (ANOVAs) with post-hoc Bonferroni tests, as well as their differences in EE measurements in each speed test. To discuss the differences in measurements of the two measurement systems used in this study, CM-EE and GT9X-EE, paired t-tests, the Cohen’s d effect size (ES), and mean absolute percentage error (MAPE) were determined for analysis. Linear regression with cross-validation (70% of samples for modeling and 30% of samples for validation) was used to modify the EE prediction model with the variables of VM and body weight (BW), and without/with HRR (Model A and Model B, respectively). The validity and reliability of the EE measurements were further evaluated using the Pearson coefficient of determination (*r*) and intraclass correlation coefficient (ICC) respectively.

## Results

**Figure 2:**
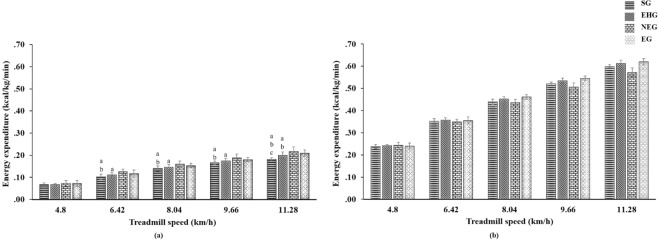
(**a**) CM-EE and **(b)** GT9X-EE (ankle-mounted) during treadmill walking and running tests at different speeds. ^a^ Significantly different from NEG, *p* < 0.001. ^b^ Significantly different from EG, *p* < 0.001. ^c^ Significantly different from EHG, *p* < 0.001. CM-EE, criterion measure’s energy expenditure. GT9X-EE, ActiGraph GT9X-Link ankle-mounted accelerometer’s energy expenditure. SG, sedentary group. EHG, exercise habit group. NEG, non-endurance group. EG, endurance group.

The ANOVA statistical data derived from the CM-EE and GT9X-EE accelerometer outputs of the four groups of different physical fitness levels (SG, EHG, NEG, and EG) are presented in Fig. 2. The results indicated a significant difference in CM-EE measurements among these four groups (*p* < 0.001). Apart from the speed of 4.8 km/h, when the CM-EE measurements showed minor differences (*p* > 0.05), the CM-EE measurements of the NEG and EG were higher than those of the SG and EHG (*p* < 0.001, *t*-test with Bonferroni correction) at the other treadmill speeds (6.4, 8.0, 9.7, 11.3 km/h). The statistical data of the GT9X-EE accelerometer output indicated that the four groups differed beyond the level of significance (*p* > 0.05). Table 2 lists the CM-EE and GT9X-EE values of the SG, EHG, NEG, and EG in exercise, and the differences in these two measurement systems (paired *t*-test), ES, MAPE, and ICC. We found that the CM-EE and GT9X-EE results of these four groups had significant differences in all speed tests (*p* < 0.001), rather high degrees of difference in ES (SG: 5.11–6.38; EHG: 5.96–6.59; NEG: 4.34–5.16; EG: 5.96–7.22) and MAPE (SG: 205.56–246.38%; EHG: 205.47–255.88%; NEG: 163.59–234.25%; EG: 195.24–228.77), and poor reliability (ICC: SG = 0.108; EHG = 0.108; NEG = 0.131; EG = 0.115).

**Table 2:** Comparison of measured EE by Vmax system (CM-EE) and estimated EE by ankle-mounted GT9X (GT9X-EE) in five treadmill walking and running tests.

Group	Treadmill Speed (km/h)	CM-EE (kcal/kg/min)	GT9X-EE (kcal/kg/min)	ES	MAPE (%)	ICC
SG	4.80	0.069 ± 0.007	0.239 ± 0.037*	6.38	246.38	0.108
	6.42	0.106 ± 0.012	0.352 ± 0.067*	5.11	232.08	
	8.04	0.144 ± 0.007	0.440 ± 0.078*	5.35	205.56	
	9.66	0.169 ± 0.011	0.522 ± 0.093*	5.33	208.88	
	11.28	0.194 ± 0.012	0.599 ± 0.105*	5.42	208.76	
EHG	4.80	0.068 ± 0.005	0.242 ± 0.037*	6.59	255.88	0.108
	6.42	0.111 ± 0.010	0.358 ± 0.057*	6.04	225.52	
	8.04	0.147 ± 0.011	0.452 ± 0.065*	6.54	207.48	
	9.66	0.168 ± 0.011	0.535 ± 0.082*	6.27	218.45	
	11.28	0.201 ± 0.014	0.614 ± 0.097*	5.96	205.47	
NEG	4.80	0.073 ± 0.013	0.244 ± 0.045*	5.16	234.25	0.131
	6.42	0.125 ± 0.012	0.349 ± 0.072*	4.34	179.20	
	8.04	0.160 ± 0.014	0.436 ± 0.086*	4.48	172.50	
	9.66	0.188 ± 0.018	0.506 ± 0.090*	4.90	169.15	
	11.28	0.217 ± 0.020	0.572 ± 0.100*	4.92	163.59	
EG	4.80	0.073 ± 0.013	0.240 ± 0.030*	7.22	228.77	0.115
	6.42	0.117 ± 0.016	0.355 ± 0.051*	6.30	203.42	
	8.04	0.154 ± 0.010	0.462 ± 0.067*	6.43	200.00	
	9.66	0.180 ± 0.010	0.545 ± 0.086*	5.96	202.78	
	11.28	0.210 ± 0.014	0.620 ± 0.092*	6.23	195.24	

Values are reported as mean ± SD. CM-EE, criterion measure energy expenditure. GT9X, ActiGraph GT9X-Link ankle-mounted accelerometer. * significantly different from CM-EE, *p* < 0.001. ES, effect size (Cohen’s *d*). Mean Absolute Percentage Error (MAPE) = {[|(predicted value – actual value)|/actual value] × 100}/*n*. ICC, intraclass correlation coefficient.

Table 3 shows the linear regression results of two prediction models formed by VM activity counts, weight, and without/with HRR (Models A and B). The linear regression results revealed that both Models A and B had high *R*^2^ values and low standard errors of estimate (SEE). The adjusted EE and CMEE results of Models A and B are listed in Table 4. Table 5 displays the Freedson VM3 Combination equation (2011). The analysis of validity (*r*) and reliability (ICC) of the EE and CMEE estimated in Models A and B among different groups indicated that the *r* (Model A: SG = 0.972, EHG = 0.938, NEG = 0.932, EG = 0.931; Model B: SG = 0.975, EHG = 0.946, NEG = 0.938, EG = 0.933) and ICC (Model A: SG = 0.954, EHG = 0.917, NEG = 0.913, EG = 0.929; Model B: SG = 0.959, EHG = 0.945, NEG = 0.932, EG = 0.930) of the modified models were both higher than those of the Freedson VM3 Combination equation (*r*: SG = 0.870, EHG = 0.869, NEG = 0.841, EG = 0.875; ICC: SG = 0.108, EHG = 0.108, NEG = 0.131, EG = 0.115).

**Table 3:** Models to predict EE (kcal/kg/min) from ankle’s VM, BW, and with/without HRR. Model A and B were developed on 70% of samples for modeling and cross-validated on the remaining 30% samples.

Model	Group	Prediction equation	*R*^2^	SEE
Model A	SG	EE = 0.000006 × VM − 0.000292 × BW + 0.007852	0.947	0.011
	EHG	EE = 0.000006 × VM − 0.000419 × BW + 0.020757	0.898	0.015
	NEG	EE = 0.000007 × VM − 0.000168 × BW − 0.005442	0.870	0.019
	EG	EE = 0.000006 × VM − 0.000170 × BW + 0.007280	0.867	0.018
Model B	SG	EE = 0.000005 × VM − 0.000271 × BW + 0.000284 × HRR + 0.012550	0.951	0.010
	EHG	EE = 0.000004 × VM − 0.000417 × BW + 0.000478 × HRR + 0.031277	0.894	0.016
	NEG	EE = 0.000005 × VM − 0.000342 × BW + 0.000542 × HRR + 0.021134	0.880	0.018
	EG	EE = 0.000005 × VM − 0.000200 × BW + 0.000304 × HRR + 0.014313	0.870	0.018

VM, vector magnitudes. BW, body weight in kg. HRR, heart rate reserve. *R*^2^, coefficient of determination. SEE, standard error of estimate.

**Table 4:** Measured EE by Vmax system (CM-EE) and estimated EE (Models A and B) by ankle-mounted GT9X in five treadmill walking and running tests (mean ± SD).

Group	Treadmill Speed (km/h)	CM-EE (kcal/kg/min)	Model A EE (kcal/kg/min)	Model B EE (kcal/kg/min)
SG	4.80	0.069 ± 0.007	0.075 ± 0.007	0.074 ± 0.006
	6.42	0.106 ± 0.012	0.116 ± 0.010	0.115 ± 0.010
	8.04	0.144 ± 0.007	0.149 ± 0.009	0.150 ± 0.009
	9.66	0.169 ± 0.011	0.178 ± 0.008	0.179 ± 0.008
	11.28	0.194 ± 0.012	0.207 ± 0.008	0.206 ± 0.008
EHG	4.80	0.068 ± 0.005	0.079 ± 0.009	0.073 ± 0.007
	6.42	0.111 ± 0.010	0.120 ± 0.011	0.111 ± 0.011
	8.04	0.147 ± 0.011	0.154 ± 0.011	0.144 ± 0.010
	9.66	0.168 ± 0.011	0.182 ± 0.013	0.172 ± 0.011
	11.28	0.201 ± 0.014	0.210 ± 0.015	0.197 ± 0.012
NEG	4.80	0.073 ± 0.013	0.087 ± 0.011	0.083 ± 0.012
	6.42	0.125 ± 0.012	0.132 ± 0.014	0.127 ± 0.012
	8.04	0.160 ± 0.014	0.169 ± 0.015	0.164 ± 0.014
	9.66	0.188 ± 0.018	0.200 ± 0.012	0.195 ± 0.012
	11.28	0.217 ± 0.020	0.229 ± 0.014	0.224 ± 0.012
EG	4.80	0.073 ± 0.013	0.080 ± 0.007	0.078 ± 0.006
	6.42	0.117 ± 0.016	0.119 ± 0.011	0.115 ± 0.010
	8.04	0.154 ± 0.010	0.155 ± 0.012	0.151 ± 0.012
	9.66	0.180 ± 0.010	0.183 ± 0.013	0.178 ± 0.013
	11.28	0.210 ± 0.014	0.208 ± 0.014	0.203 ± 0.014

**Table 5:** Validity and reliability analysis of the predicted EE in models and CMEE in different groups.

Group	Freedson VM3 Combination equation		Model A		Model B	
	*r*	ICC	*r*	ICC	*r*	ICC
SG	0.870	0.108	0.972	0.954	0.975	0.959
EHG	0.869	0.108	0.938	0.917	0.946	0.945
NEG	0.871	0.131	0.932	0.913	0.938	0.932
EG	0.845	0.115	0.931	0.929	0.933	0.930

*r*, Pearson coefficient of determination. ICC, intraclass correlation coefficient.

## Discussion

We employed indirect calorimetry and ankle-mounted accelerometer outputs to examine the EE estimation of groups with different physical fitness and to establish an appropriate prediction equation. The cross-validation results showed that the differences in correlation in four groups between 70% and 30% were from 0.000 to 0.008, and all correlations were higher than 0.913. This means that the new EE prediction equations developed by this research have a high degree of utility. The EE measurement results of ankle-mounted accelerometer outputs were unlikely to identify different groups of participants, and, compared with the CMEE, over-estimation was found (*p* < 0.001). We employed the Freedson VM3 Combination equation (2011)^32^, and the measurement results indicated the impossibility of identifying differences in the EEs of different groups from the data collected from ankle-mounted devices due to the high overestimation value of 200% and poor predictive ability (SG: MAPE = 205.56–246.38%, ICC = 0.108; EHG: MAPE = 205.47–255.88%, ICC = 0.108; NEG: MAPE = 163.59–234.25%, ICC = 0.131; EG: MAPE = 195.24–228.77%, ICC = 0.115). In our previous study^30^, we found that including HRR parameters in the prediction equation could calibrate for individuals’ physical fitness and standardize an individual’s physical fitness level, helping to increase the accuracy of EE estimates via a wearable device. This phenomenon was also found in this study. Similarly, after the inclusion of the HRR parameters in the prediction equation (Model B), the validity and reliability of the four groups’ EE estimate considerably improved, but such improvement was not significant compared with that of Model A (where the coefficient of the Freedson VM3 Combination equation (2011)^32^ was modified).

Physical exercise is accomplished by the contraction and relaxation of skeletal muscles. However, the contraction and relaxation of muscles function normally only when the energy supplied to them is sufficient. The larger the muscle mass, the more energy is consumed. We also found that an athlete’s muscle mass (NEG: 32.1 ± 7.1 kg, EG: 29.1 ± 4.5 kg) was greater than that of a non-athlete (SG: 26.6 ± 5.7 kg, EHG: 28.4 ± 6.0 kg) (*p* < 0.05), the EE of the athlete group during exercise was also higher than that of the non-athlete group (*p* < 0.05), and a similar pattern was noted in the RMRs of the different groups. As a result, when the treadmill speed (or exercise intensity) was the same, the CMEE indicated that when the EEs of two athlete groups were higher than those of non-athlete groups, the EE of the NEG was the highest and could change according to the treadmill speed (or exercise intensity) following the same pattern. This result is identical to that of a previous study indicating that the RMR and TEE of athletes are higher than those of non-athlete groups^3,6,35,38,39^. Petridou *et al*. discussed the TEE of 14 male endurance athletes and non-athletes and found that the TEEs of athletes and non-athletes were 3895 ± 600 kcal/day and 2722 ± 475 kcal/day (*p* < 0.05) respectively, and the REE of athletes was also higher than that of non-athletes (1407.3 ± 170.2 kcal/day, and 1259.1 ± 105.6 kcal/day, respectively)^40^. Ndahimana *et al*. analyzed the TEE of college-level tennis players and non-athletes (aged 19 to 24 years) and reported that the TEEs of tennis players and non-athletes were 2780.3 ± 429.5 kcal/day and 2012.3 ± 160.5 kcal/day (*p* = 0.001), respectively^3^. Matsushita *et al*. discussed the TEE of 34 female rhythmic gymnasts athletes and 23 female students without exercise habits (non-athletes) and found that the TEE of athletes and non-athletes were 2317 ± 261 kcal/day and 1907 ± 204 kcal/day (*p* < 0.001), respectively^39^.

Accelerometer-based physical activity monitoring devices estimate the exercise intensity, EE, and other physical activity parameters based on VM activity counts produced during the exercise. Due to the limits of such devices, the generated VM activity counts can vary when the wearing position changes. In other words, with the same exercise intensity, VM activity counts can increase or decrease due to different device-wearing positions and result in bias in EE estimates compared with standard measurements. In this study, the GT9X EE measurements at all speed levels in all four groups were overestimated when an ankle-mounted device was worn (*p* < 0.001), and a large difference in effect size (4.34 to 7.22) was found. The EE prediction equation currently applicable to the accelerometer device is designed for healthy adult participants, and the device is worn on the waist to collect activity parameters to develop an estimation equation using a linear regression method^19,20^. Our results indicate that although the GT9X EE and standard measurements of ankle-mounted devices demonstrated poor reliability at all speed levels (SG: ICC = 0.108; EHG: ICC = 0.108; NEG: ICC = 0.131; EG: ICC = 0.115), the GT9X EE and CMEE measurements of each group had high validity (SG: *r* = 0.870; EHG: *r* = 0.869; NEG: *r* = 0.871; EG: *r* = 0.845). Therefore, although the EE and CMEE estimated by ankle-mounted devices have high validity, they have poor reliability due to the characteristics of VM activity counts. To avoid bias, an appropriate EE estimation equation is required when the different vibration counts caused by wearing location are not considered.

To improve the limits of accelerometer sensing devices, some researchers have proposed combining the activity parameters of accelerometers with HR parameters to increase the accuracy of EE estimates^29,41–43^. Romero-Ugalde *et al*. highlighted the importance of activity EE in health care and the necessity of having accurate and efficient EE measures. The EE estimation equation developed through the combination of heart rate and waist-mounted accelerometer signals (*R*^2^ = 0.8385, root mean square error (RMSE) = 59.3617 J/kg/min) can be used accurately estimate the EE of light to moderate physical activity (binary classification accuracy of 0.8155)^44^. Chang *et al*. increased the EE estimate accuracy of an accelerometer-based monitoring device during uphill walking and running by modifying the traditional empirical equation of accelerometers using HR and HRR as compensation factors. The results indicated that compared with HR, the combination of HRR and accelerometer parameters provides better predictive ability^30^. As already reported in the literature, heart rate measurements from wearable devices can also be affected by estimation errors. This could affect the reliability of the improved estimation equations^45^. We found that both Models A and B managed to increase the validity and reliability of the ankle-mounted device’s EE measurements in all four groups, with the estimates close to the real values. The MAPE of Model A and standard measurements were divided into SG: 6.95%, EHG: 8.24%, NEG: 8.46% and EG: 2.91%; the MAPE of Model B and standard measurements were divided into SG: 6.40%, EHG: 2.62%, NEG: 4.95% and EG: 2.99%. However, we observed no significant differences between Model A (*r* = 0.931 to 0.972, ICC = 0.913 to 0.954) and Model B (*r* = 0.933 to 0.975, ICC = 0.930 to 0.960). This means that the accuracy of the two models is approximately equal, either by modifying the coefficients of traditional estimation equations (Model A) or adding HRR parameters to traditional estimation equations (Model B).

During walking and running, the ankle joint is the first body part that suffers from ground reaction force and impact force^46^. This is the same when wearable devices are used; the ankle-mounted device suffers from maximum impact force, which results in the highest VM activity counts. Hibbing *et al*. reported that, similarly, when 10 activities ranging from rest to running were performed when ActiGraph GT9X accelerometers were mounted on the right hip, both wrists, and both ankles simultaneously, the VM activity counts of the ankle-mounted device were higher than those of devices worn in other positions^47^. If the traditional estimation equation (Freedson VM3 Combination equation, 2011)^32^, which is mainly used for the waist, is used with the ankle-mounted device, overestimation can also result. It can be seen that it is essential to modify the coefficient of the estimation equation, or modified/developed new estimation equations, which can correct the bias caused by different wearing positions. In summary, in the treadmill walking/running tests conducted in this study, the Actigraph worn on the ankle led to overestimated EE measurements. However, the estimation accuracy can be improved by modifying the coefficient of the estimation equation (Model A) or combining HRR parameters with the estimation equation (Model B). Model A (without HRR) seems to be the best choice for EE estimation as it is the simplest and most reasonable method.

In conclusion, as ankle-mounted activity monitoring devices provide relatively higher VM activity counts, and the EE estimation accuracy is therefore affected. By modifying the coefficient of the Freedson VM3 Combination equation (2011) (Model A) and including HRR parameters in the estimation equation (Model B), we enhanced the EE estimation accuracy. The rapid progress of technology has simplified data collection by using the simplest method to obtain the most accurate estimates. We found that Model A is the better model for measuring the EE estimates of ankle-mounted devices. Since the participants in this study were mainly healthy adult university students and members of university sports teams, the equation that we developed in this study may not be applicable to other groups, such as pre-school children, adolescents, the elderly, or people with specific diseases or physical difficulties.
